# Catalytic Hydrolysis of Phosphate Monoester by Supramolecular Complexes Formed by the Self-Assembly of a Hydrophobic Bis(Zn^2+^-cyclen) Complex, Copper, and Barbital Units That Are Functionalized with Amino Acids in a Two-Phase Solvent System

**DOI:** 10.3390/mi10070452

**Published:** 2019-07-04

**Authors:** Yuya Miyazawa, Akib Bin Rahman, Yutaka Saga, Hiroki Imafuku, Yosuke Hisamatsu, Shin Aoki

**Affiliations:** 1 Faculty of Pharmaceutical Sciences, Tokyo University of Science, 2641 Yamazaki, Noda, Chiba 278-8510, Japan; 2 Research Institute for Science and Technology, Tokyo University of Science, 2641 Yamazaki, Noda, Chiba 278-8510, Japan

**Keywords:** supramolecular chemistry, zinc, copper, hydrolysis, dephosphorylation, self-assembly

## Abstract

We previously reported on the preparation of supramolecular complexes by the 2:2:2 assembly of a dinuclear Zn^2+^-cyclen (cyclen = 1,4,7,10-tetraazacyclododecane) complex having a 2,2′-bipyridyl linker equipped with 0~2 long alkyl chains (Zn_2_L^1^~Zn_2_L^3^), 5,5-diethylbarbituric acid (Bar) derivatives, and a copper(II) ion (Cu^2+^) in aqueous solution and two-phase solvent systems and their phosphatase activities for the hydrolysis of mono(4-nitrophenyl) phosphate (MNP). These supermolecules contain Cu_2_(*μ*-OH)_2_ core that mimics the active site of alkaline phosphatase (AP), and one of the ethyl groups of the barbital moiety is located in close proximity to the Cu_2_(*μ*-OH)_2_ core. The generally accepted knowledge that the amino acids around the metal center in the active site of AP play important roles in its hydrolytic activity inspired us to modify the side chain of Bar with various functional groups in an attempt to mimic the active site of AP in the artificial system, especially in two-phase solvent system. In this paper, we report on the design and synthesis of new supramolecular complexes that are prepared by the combined use of bis(Zn^2+^-cyclen) complexes (Zn_2_L^1^, Zn_2_L^2^, and Zn_2_L^3^), Cu^2+^, and Bar derivatives containing amino acid residues. We present successful formation of these artificial AP mimics with respect to the kinetics of the MNP hydrolysis obeying Michaelis–Menten scheme in aqueous solution and a two-phase solvent system and to the mode of the product inhibition by inorganic phosphate.

## 1. Introduction

The regulation of protein phosphorylation and dephosphorylation is an important process in terms of cellular signal transduction, apoptosis, and the cell cycle among other issues. The phosphorylation of proteins is mediated by protein kinases, such as tyrosine kinases which are related to signal transduction pathways in living cells [1]. Indeed, several molecular targeted drugs that target tyrosine kinases in cancer cells and inhibit their action have been developed for the treatment of cancer [2,3]. On the other hand, dephosphorylation is promoted by the action of protein phosphatases such as alkaline phosphatase (AP) which contains two Zn^2+^ ions in its active center [4,5,6,7,8,9,10]. Although some artificial phosphatases that mimic the active center of metallophosphatases have been reported [11,12,13,14,15,16,17,18,19,20,21,22], very few of them function as catalysts for the hydrolysis of a phosphate monoester such as mono(4-nitrophenyl) phosphate (MNP). Hence, dephosphorylation by artificial catalysts that mimic protein phosphatases remains a great challenge.

It has been described that chemically synthesized enzyme models have several disadvantages including, (i) a lack of long-range interactions between catalysts and substrate and/or between functional groups in their active sites, (ii) the synthesis of such compounds is time-consuming, and (iii) they are active only in organic solvents in many cases [23]. Indeed, the synthesis of artificial enzyme models that are constructed by covalent bonds require long and complex synthetic routes especially for their functionalization, as a result, only a limited number of structures have been reported. These drawbacks, however, could be overcome by a supramolecular strategy utilizing the self-assembly of the artificial and functionalized molecular building blocks that are readily available [24,25,26,27,28,29,30,31,32,33,34,35,36,37,38,39,40,41,42,43,44,45,46,47,48,49,50,51,52].

**Scheme 1:**
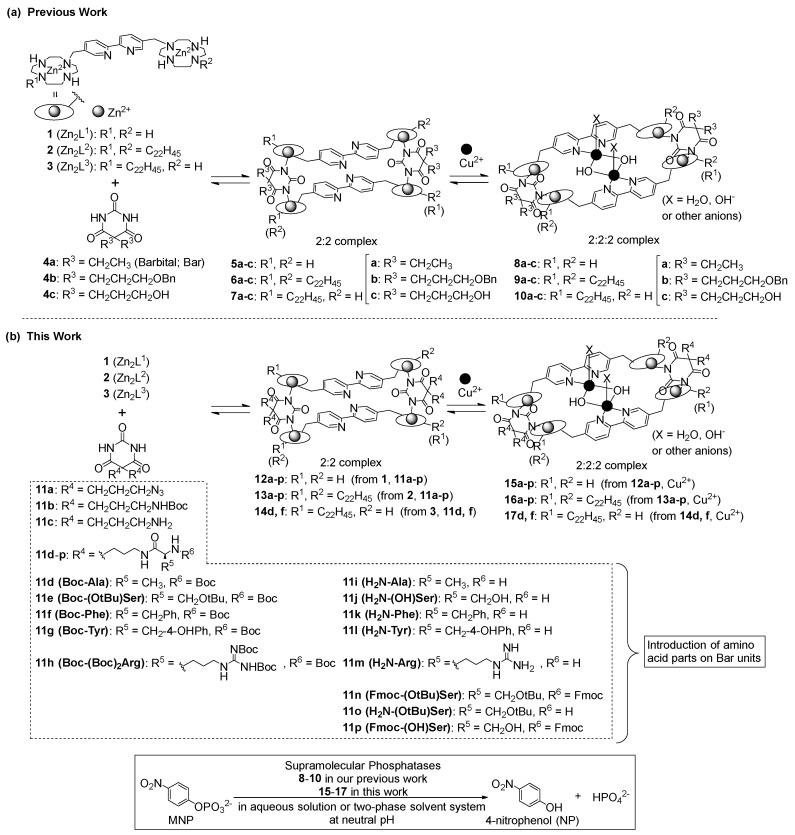
(**a**) Formation of the 2:2:2 supramolecular complexes **8**–**10** by the self-assembly of Zn_2_L complexes (**1**–**3**), 5,5-diethylbarbital (Bar) derivatives (**4a**–**c**) and Cu^2+^ in an aqueous solution at neutral pH or in a two-phase solvent system (CHCl_3_/H_2_O) as was used in our previous study. (**b**) Formation of the 2:2:2 supramolecular complexes **15**–**17** by a self-assembly of Zn_2_L complexes (**1**–**3**), 5,5-diethylbarbital (Bar) derivatives (**11a**–**p**) and Cu^2+^ in an aqueous solution at neutral pH or a two-phase solvent system (CHCl_3_/H_2_O) in this work.

In this context, we previously reported on the formation of the supramolecular complex **8a**, by the 2:2:2 assembly of a bis(Zn^2+^-cyclen) complex (cyclen = 1,4,7,10-tetraazacyclododecane) containing 2,2′-bipyridyl (bpy) linker **1** (Zn_2_L^1^), a dianion of barbital **4a** (Bar), and a copper(II) ion in an aqueous solution which is stabilized by non-covalent bonds (Scheme 1a) [19,24]. The X-ray crystal structure of the supermolecule **8a** revealed that it contains a Cu_2_(*μ*-OH)_2_ core, which resembles the active centers of metallophosphatases such as AP, and that one of the ethyl groups of the Bar unit is located in close proximity to the Cu_2_(*μ*-OH)_2_ core [24]. More importantly, **8a** accelerates the hydrolysis of MNP, although the yield is low, possibly due to product inhibition by inorganic phosphate (HPO_4_^2−^), a byproduct of the MNP hydrolysis. 

**Scheme 2:**
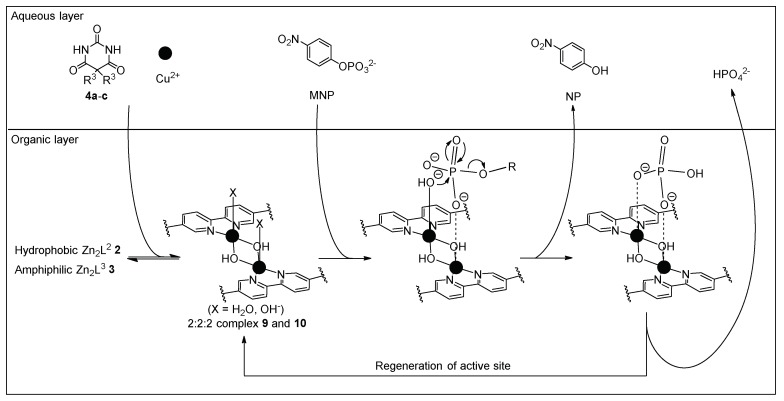
Concept of the hydrophobic supramolecular complexes **9** and **10** and the hydrolysis of mono(4-nitrophenyl) phosphate (MNP) in a two-phase solvent system, in which the product inhibition by HPO_4_^2−^ is avoided.

We also reported on the formation of the hydrophobic supramolecular complexes **9a**–**c** and the amphiphilic supramolecular complexes **10a**–**c** (Scheme 1a) by the 2:2:2 assembly of a hydrophobic bis(Zn^2+^-cyclen) complex containing two long alkyl chains **2** (Zn_2_L^2^) and a complex that contained one long alkyl chain **3** (Zn_2_L^3^), respectively, with Bar^2−^ derivatives and Cu^2+^ in a two-phase solvent system (CHCl_3_/H_2_O) [53,54]. As shown in Scheme 2, we expected that the hydrophobic or amphiphilic 2:2:2 complexes **9** or **10** would be formed mainly in the organic layer, and that product inhibition by HPO_4_^2−^ could be avoided, since the hydrophilic HPO_4_^2−^ would be released into the aqueous layer, thus permitting the Cu_2_(*μ*-OH)_2_ core to be regenerated. The results showed that **9** and **10** accelerated the hydrolysis of MNP and one of most important findings was that the hydrolysis of MNP by **9** and **10** in the two-phase solvent system obeyed Michaelis–Menten kinetics, suggesting that these reaction systems consisting of a supramolecular complex in two-phase solvent system closely mimic the active sites of AP. However, catalytic activity was observed only in the case of **10** (the catalytic turnover numbers (CTN) were 3~4 [54]), but not in **9** [53]. In addition, the product inhibition of **9** and **10** by inorganic phosphate (HPO_4_^2−^) was not competitive, although it is well known that HPO_4_^2−^ inhibits natural AP in a competitive manner. These results raised next question of how to design and synthesize good and appropriate models for natural enzymes such as AP based on a supramolecular strategy.

**Scheme 3:**
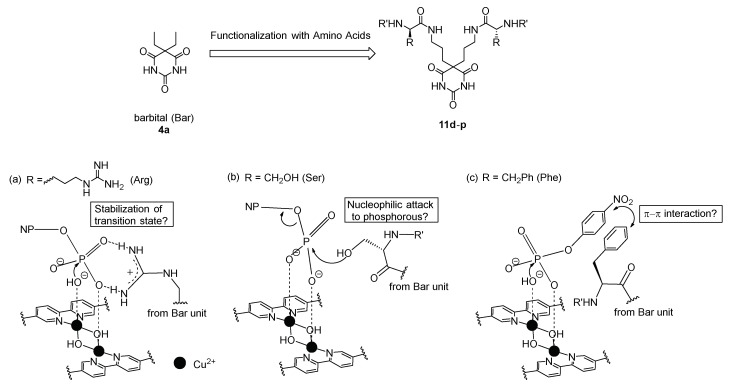
Predicted effect of new barbital derivatives functionalized with amino acids on the hydrolysis of MNP. (**a**) It is considered that Arg stabilizes the transition state of the MNP hydrolysis by the electrostatic interactions between them, (**b**) Ser is supposed to undergo the nucleophilic attack to the phosphorous of MNP, (**c**) Phe is supposed to contribute to the π–π interactions with MNP.

It is generally accepted that amino acids in the active site of AP contribute to the hydrolysis of phosphomonoesters; for example, the arginine residue captures and fixes the conformation of the phosphomonoester, and the serine residue attacks the phosphorous to promote the hydrolysis [4,5,6,7,8,9,10]. These well-grounded assumptions prompted us to introduce amino acids (alanine, serine, arginine, phenylalanine, and tyrosine) into the side chains of the Bar units for the easy and versatile construction of a combinatorial library of supramolecular phosphatases (Scheme 3). For example, functionalization with arginine (Arg) unit would be expected to stabilize the transition state that is produced in the hydrolysis of MNP (Scheme 3a) by electrostatic interactions. Second, the serine (Ser) residue would participate in a nucleophilic attack on the phosphorous of the MNP (Scheme 3b). Third, a phenylalanine (Phe) unit was introduced to support π–π interactions with MNP for a stronger complexation (Scheme 3c). In this paper, we report on the combinatorial construction of 2:2:2 supramolecular complexes **15**–**17** from dinuclear Zn^2+^-cyclen complexes **1** (Zn_2_L^1^), **2** (Zn_2_L^2^), or **3** (Zn_2_L^3^) with the Bar derivatives that are functionalized with amino acids **11d**–**p** as well as their synthetic intermediates **11a**–**c** and Cu^2+^ (Scheme 1b), their catalytic activity for the hydrolysis of MNP, and the results of a Michaelis–Menten kinetic study. These findings indicate that hydrophobic active sites of the supramolecular phosphatases well mimic the mode of the substrate (MNP) recognition and the product inhibition (by inorganic phosphate), and both of hydrophobic and hydrophilic property of active sites are important for the catalytic turnover of the system.

## 2. Materials and Methods

### 2.1. General Information

All reagents and solvents were of the highest commercial quality and were used without further purification, unless otherwise noted. Anhydrous *N*,*N*-dimethylformamide (DMF) was obtained by distillation from calcium hydride. MNP was purchased from Nacalai Tesque (Kyoto, Japan). All aqueous solutions were prepared using deionized and distilled water. The Good’s buffer reagents (Dojindo, p*K*_a_ at 20 °C) were obtained from commercial sources: HEPES (2-(4-(2-hydroxyethyl)-1-piperazinyl))ethanesulfonic acid, p*K*_a_ = 7.6). For the measurement of UV/Vis spectra, CHCl_3_ was purchased from Nacalai Tesque (Kyoto, Japan). UV/Vis spectra were recorded on a JASCO V-550 spectrophotometer with quartz cuvettes (path length: 10 mm). IR spectra were recorded on a Perkin-Elmer attenuated total reflectance (ATR)-IR spectrometer 100 at room temperature. Melting points were measured on a Yanaco MP-J3 Micro Melting Point apparatus and are uncorrected. ^1^H- (300 and 400 MHz) and ^13^C- (75 and 100 MHz) NMR spectra at 25 ± 0.1 °C were recorded on a JEOL Always 300 spectrometer and a JEOL Lamda 400 spectrometer. Tetramethylsilane (TMS) was used as the internal reference for ^1^H- and ^13^C-NMR measurements in CDCl_3_ and CD_3_OD. 3-(Trimethylsilyl)propionic-2,2,3,3-*d_4_* acid sodium salt (TSP) was used as the external reference for ^1^H- and ^13^C-NMR measurements in D_2_O. Mass spectra was recorded on a JEOL JMS-700 and Varian 910-MS spectrometer. Elemental analyses were performed on a Perkin-Elmer CHN 2400 analyzer. Optical rotations were measured with a JASCO-P-1030 digital polarimeter in 50 mm cells using the D line of sodium (589 nm). Thin-layer chromatography (TLC) and silica gel column chromatography was performed using Merck Silica gel 60 F254 plate or Fuji Silysia Chemical CHROMATOREX NH-TLC PRATE, and Fuji Silysia Chemical FL-100D or Fuji Silysia Chemical CHROMATOREX NH chromatography Silica Gel, respectively.

### 2.2. Synthesis of Compounds

5,5-Bis[3-(toluenesulfonyl)oxypropyl]barbituric acid (**18**)

A solution of **4c** (698 mg, 2.86 mmol) and DMAP (35 mg, 0.29 mmol) in pyridine (5 mL) was stirred at 0 °C for 5 min, to which *p*-toluenesulfonyl chloride (1.53 g, 8.00 mmol) was slowly added, and the resulting reaction mixture was stirred at room temperature for 2 h. The reaction was quenched by adding an aqueous solution saturated with NaHCO_3_, and the resulting suspension was extracted with CHCl_3_ (50 mL × 3). The combined organic layer was washed with brine, dried over Na_2_SO_4_, and concentrated under reduced pressure. The resulting residue was purified by silica gel column chromatography (CHCl_3_/MeOH = 1/0 to 50/1) to give **18** as a colorless amorphous solid (819 mg, 54% yield). ^1^H NMR (300 MHz, CDCl_3_, TMS): *δ* = 7.75 (d, *J* = 8.3 Hz, 4H), 7.35 (d, *J* = 8.3 Hz, 4H), 3.98–3.92 (m, 4H), 2.45 (s, 6H), 2.12–1.94 (m, 4H), 1.68–1.56 (m, 4H) ppm; ^13^C NMR (75 MHz, CDCl_3_, TMS): *δ* = 171.7, 149.0, 145.1, 132.5, 129.9, 127.9, 69.4, 43.8, 34.0, 24.3, 21.6 ppm; IR (ATR): *ν* = 3232, 3106, 2962, 2856, 1756, 1696, 1596, 1419, 1346, 1171, 1096, 953, 914, 811, 660, 575, 552, 493. HRMS (ESI): *m/z* calcd for [M+Na]^+^, C_24_H_28_N_2_O_9_NaS_2_, 575.1124; found, 575.1129.

5,5-Bis(3-azidopropyl)barbituric acid (**11a**)

A solution of **18** (850 mg, 1.54 mmol) and sodium azide (280 mg, 4.31 mmol) in DMF (4 mL) was stirred at 80 °C for 5 h. After allowing the solution to cool to room temperature, the reaction mixture was poured into H_2_O and the resulting mixture was extracted with Et_2_O (40 mL × 3). The combined organic layer was washed with brine, dried over Na_2_SO_4_, and concentrated under reduced pressure. The resulting residue was purified by silica gel column chromatography (CHCl_3_/MeOH = 1/0 to 10/1) to give **11a** as a white solid (434 mg, 96% yield). ^1^H NMR (300 MHz, CDCl_3_, TMS): *δ* = 8.71 (s, 2H), 3.29 (t, *J* = 6.6 Hz, 4H), 2.11–2.05 (m, 4H), 1.59–1.49 (m, 4H) ppm; ^13^C NMR (75 MHz, CDCl_3_, TMS): *δ* = 171.5, 55.5, 50.7, 35.6, 24.5 ppm; mp 118–119 °C; IR (ATR): *ν* = 3198, 3090, 2940, 2862, 2093, 1752, 1694, 1417, 1361, 1326, 1273, 1248, 1199, 1132, 1043, 835, 674, 538, 496. HRMS (ESI): *m/z* calcd for [M-H]^−^, C_10_H_13_N_8_O_3_, 293.1122; found, 293.1116. Anal. Calcd. for C_10_H_14_N_8_O_3_: C, 40.82; H, 4.80; N, 38.08; found: C, 40.91; H, 4.74; N, 37.79.

5,5-Bis[3-(*t*-butoxycarbonylamino)propyl]barbituric acid (**11b**)

Solutions of **11a** (420 mg, 1.43 mmol) in AcOEt (2 mL) and Boc_2_O (760 mg, 3.48 mmol) in AcOEt (2 mL) were added to a suspension of 10% Pd/C (50 mg) in AcOEt (2 mL) at room temperature under a N_2_ atmosphere. The reaction mixture was hydrogenated with H_2_ (at 1 atm) at room temperature for 24 h. After the reaction reached completed, the reaction mixture was filtered, and the filtrate was evaporated under reduced pressure. The resulting residue was purified by silica gel column chromatography (AcOEt/Hexanes = 2/3) to give **11b** as a colorless amorphous solid (440 mg, 71% yield). ^1^H NMR (300 MHz, CDCl_3_, TMS): *δ* = 9.32 (brs, 2H), 4.78–4.68 (m, 2H), 3.12–3.00 (m, 4H), 2.01–1.96 (m, 4H), 1.47–1.40 (m, 22H) ppm; ^13^C NMR (75 MHz, CDCl_3_, TMS): *δ* = 172.7, 156.0, 150.0, 79.4, 55.5, 39.9, 35.7, 28.3, 25.4 ppm; IR (ATR): *ν* = 3357, 3203, 3090, 2977, 2935, 1755, 1686, 1516, 1448, 1392, 1365, 1275, 1248, 1163, 1040, 854, 756, 666, 494. HRMS (ESI): *m/z* calcd for [M+H]^+^, C_20_H_35_N_4_O_7_, 443.2501; found, 443.2500.

5,5-Bis(3-aminopropyl)barbituric acid·2HCl salt (**11c**)

Compound **11b** (440 mg, 0.99 mmol) was dissolved in MeOH (2 mL), to which conc. HCl (2 mL) was added at 0 °C. The mixture was stirred at room temperature for 1 h and concentrated under reduced pressure to give **11c** as a white solid (276 mg, 86% yield as the 2HCl salt). ^1^H NMR (300 MHz, CD_3_OD, TMS): *δ* = 2.90 (t, *J* = 7.2 Hz, 4H), 2.05–2.00 (m, 4H), 1.66–1.56 (m, 4H) ppm; ^13^C NMR (100 MHz, D_2_O, TSP): *δ* = 173.8, 150.5, 55.0, 38.9, 34.2, 22.3 ppm; mp 268 °C; IR (ATR): *ν* = 2967, 2826, 1749, 1721, 1694, 1591, 1485, 1443, 1397, 1346, 1283, 1220, 981, 819, 492. HRMS (ESI): *m/z* calcd for [M+H]^+^, C_10_H_19_N_4_O_3_, 243.1456; found, 243.1452. Anal. Calcd. for C_10_H_20_N_4_O_3_Cl_2_: C, 38.11; H, 6.40; N, 17.78; found: C, 38.31; H, 6.42; N, 17.85.

5,5-Bis{3-[*N*-(*t*-butoxycarbonyl)-l-alanyl]aminopropyl}barbituric acid (**11d**)

To a suspension of **11c** (10 mg, 0.03 mmol) in anhydrous DMF (0.5 mL) and diisopropylethylamine (22 mg, 0.17 mmol), Boc-l-Alanine-OH (13 mg, 0.07 mmol) and PyBOP (36 mg, 0.07 mol) were added and the reaction mixture was stirred at room temperature for 1 h. The reaction mixture was concentrated under reduced pressure, dissolved in CHCl_3_ (10 mL), washed with a 50% NH_4_Cl solution (10 mL × 3), then with brine, dried over Na_2_SO_4_ and concentrated under reduced pressure. The resulting oil was purified by silica gel column chromatography (AcOEt/Hexanes = 1/2) and reprecipitation with AcOEt and hexanes to give **11d** as a white solid (17 mg, 92% yield). ^1^H NMR (300 MHz, CDCl_3_, TMS): *δ* = 10.2 (brs, 2H), 7.06 (brs, 2H), 5.62–5.58 (m, 2H), 4.22 (brs, 2H), 3.17–3.14 (m, 4H), 1.96–1.92 (m, 4H), 1.45–1.40 (m, 22H), 1.33 (d, *J* = 5.1 Hz, 6H) ppm; ^13^C NMR (75 MHz, CDCl_3_, TMS): *δ* = 173.5, 172.8, 155.8, 149.9, 55.4, 50.0, 46.3, 39.0, 35.7, 28.4, 24.8, 18.8 ppm; mp 108–109 °C; IR (ATR): *ν* = 3315, 3092, 2977, 2935, 1754, 1692, 1653, 1522, 1448, 1365, 1246, 1162, 1055, 1021, 856, 759, 495. HRMS (ESI): *m/z* calcd for [M+H]^+^, C_26_H_45_N_6_O_9_, 585.3245; found, 585.3243. Anal. Calcd. for C_26_H_44_N_6_O_9_·0.5H_2_O: C, 52.60; H, 7.64; N, 14.16; found: C, 52.51; H, 7.45; N, 14.20. [α]^25^_D_ = −17.9 (*c* = 1.0, CHCl_3_).

5,5-Bis{3-[*N*-(*t*-butoxycarbonyl)-*O*-(*t*-butyl)-l-seryl]aminopropyl}barbituric acid (**11e**)

Compound **11e** was synthesized as a white solid (29 mg, 84% yield) from **11c** (10 mg, 0.03 mmol), Boc-l-Serine(OtBu)-OH (18 mg, 0.07 mmol), diisopropylethylamine (22 mg, 0.17 mmol), and PyBOP (36 mg, 0.07 mmol) in anhydrous DMF (0.5 mL) using a procedure similar to that used for **11d**. ^1^H NMR (300 MHz, CDCl_3_, TMS): *δ* = 9.27 (brs, 2H), 6.71 (t, *J* = 6.0 Hz, 2H), 5.47 (d, *J* = 6.9 Hz, 2H), 4.20–4.10 (m, 2H), 3.80–3.76 (m, 2H), 3.42–3.36 (m, 2H), 3.30–3.10 (m, 4H), 2.03–1.92 (m, 4H), 1.48–1.39 (m, 22H), 1.18 (s, 18H) ppm; ^13^C NMR (100 MHz, CDCl_3_, TMS): *δ* = 172.2, 171.0, 155.7, 148.8, 80.1, 74.0, 61.8, 55.6, 54.4, 39.0, 35.8, 28.3, 27.4, 25.0 ppm; mp 136–139 °C; IR (ATR): *ν* = 3332, 3087, 2974, 2934, 2871, 1754, 1697, 1655, 1498, 1390, 1363, 1243, 1163, 1085, 1019, 861, 755, 668, 494. HRMS (ESI): *m/z* calcd for [M+H]^+^, C_34_H_61_N_6_O_11_, 729.4387; found, 729.4393. Anal. Calcd. for C_34_H_60_N_6_O_11_·H_2_O: C, 54.68; H, 8.37; N, 11.25; found: C, 54.82; H, 8.43; N, 11.20. [α]^25^_D_ = +16.7 (*c* = 1.0, CHCl_3_).

5,5-Bis{3-[N-(*t*-butoxycarbonyl)-l-phenylalanyl]aminopropyl}barbituric acid (**11f**)

Compound **11f** was synthesized as a white solid (20 mg, 86% yield) from **11c** (10 mg, 0.03 mmol), Boc-l-Phenylalanine-OH (19 mg, 0.07 mmol), diisopropylethylamine (22 mg, 0.17 mmol), and PyBOP (36 mg, 0.07 mmol) in anhydrous DMF (0.5 mL) using a procedure similar to that used for **11d**. ^1^H NMR (300 MHz, CDCl_3_, TMS): *δ* = 9.43 (brs, 2H), 7.24–7.16 (m, 10H), 6.36–6.26 (m, 2H), 5.32 (d, *J* = 8.7 Hz, 2H), 4.39–4.30 (m, 2H), 3.20–2.98 (m, 8H), 1.90–1.78 (m, 4H), 1.38 (s, 18H), 1.38–1.24 (m, 4H) ppm; ^13^C NMR (100 MHz, CDCl_3_, TMS): *δ* = 172.5, 171.8, 155.8, 149.5, 136.8, 129.3, 128.5, 126.8, 80.2, 55.9, 55.4, 38.9, 35.5, 28.3, 24.6 ppm; mp 117–118 °C; IR (ATR): *ν* = 3319, 3088, 2978, 2933, 2859, 1695, 1655, 1526, 1497, 1366, 1249, 1165, 1023, 858, 751, 699, 495. HRMS (ESI): *m/z* calcd for [M+H]^+^, C_38_H_53_N_6_O_9_, 737.3869; found, 737.3869. Anal. Calcd. for C_38_H_52_N_6_O_9_·0.5H_2_O: C, 61.19; H, 7.16; N, 11.27; found: C, 61.18; H, 7.18; N, 11.26. [α]^25^_D_ = +2.73 (*c* = 1.0, CHCl_3_).

5,5-Bis{3-[*N*-(*t*-butoxycarbonyl)-l-tyrosyl]aminopropyl}barbituric acid (**11g**)

Compound **11g** was synthesized as a white solid (18 mg, 72% yield) from **11c** (10 mg, 0.03 mmol), Boc-l-Tyrosine-OH (20 mg, 0.07 mmol), diisopropylethylamine (22 mg, 0.17 mmol), and PyBOP (36 mg, 0.07 mmol) in anhydrous DMF (0.5 mL) using a procedure similar to that used for **11d**. ^1^H NMR (300 MHz, CD_3_OD, TMS): *δ* = 7.02 (d, *J* = 8.7 Hz, 4H), 6.70 (d, *J* = 8.7 Hz, 4H), 4.18–4.04 (m, 2H), 3.13–3.03 (m, 4H), 2.92 (dd, *J* = 3.6 Hz, 2H), 2.76–2.64 (m, 2H), 1.88–1.74 (m, 4H), 1.41–1.28 (m, 4H), 1.37 (s, 18H) ppm; ^13^C NMR (100 MHz, CD_3_OD, TMS): *δ* = 174.5, 157.5, 157.2, 151.2, 131.3, 129.2, 116.2, 80.6, 57.8, 56.5, 40.1, 40.0, 38.8, 37.0, 28.7, 25.8 ppm; mp 150–152 °C; IR (ATR): *ν* = 3320, 2975, 2930, 1693, 1651, 1515, 1446, 1366, 1244, 1160, 1104, 1019, 829, 779, 494. HRMS (ESI): *m/z* calcd for [M+H]^+^, C_38_H_53_N_6_O_11_, 769.3755; found, 769.3767. Anal. Calcd. for C_38_H_52_N_6_O_11_·2.75H_2_O: C, 55.77; H, 7.08; N, 10.27; found: C, 55.49; H, 6.58; N, 10.80. [α]^25^_D_ = +3.63 (*c* = 1.0, CH_3_OH).

5,5-Bis{3-[*N*^α^,*N*^ω^,*N*^δ^-tri(*t*-butoxycarbonyl)-l-arginyl]aminopropyl}barbituric acid (**11h**)

Compound **11h** was synthesized as a white solid (30 mg, 82% yield) from **11c** (10 mg, 0.03 mmol), Boc-l-Arginine(Boc)_2_-OH (35 mg, 0.07 mmol), diisopropylethylamine (22 mg, 0.17 mmol), and PyBOP (36 mg, 0.07 mmol) in anhydrous DMF (0.5 mL) using a procedure similar to that used for **11d**. ^1^H NMR (300 MHz, CDCl_3_, TMS): *δ* = 9.33 (brs, 2H), 7.14–7.08 (m, 2H), 6.00–5.92 (m, 2H), 4.32–4.22 (m, 2H), 4.04–3.90 (m, 2H), 3.73–3.60 (m, 2H), 3.35–3.05 (m, 4H), 1.94–1.82 (m, 4H), 1.82–1.74 (m, 4H), 1.52 (s, 18H), 1.48 (s, 18H), 1.47–1.30 (m, 12H), 1.44 (s, 18H) ppm; ^13^C NMR (100 MHz, CDCl_3_, TMS): *δ* = 172.3, 171.9, 163.2, 161.0, 155.7, 155.0, 148.5, 84.2, 79.8, 79.4, 55.5, 53.7, 44.0, 39.1, 36.1, 28.9, 28.5, 28.4, 28.1, 25.1, 24.5 ppm; mp 123–125 °C; IR (ATR): *ν* = 3380, 2977, 2934, 1755, 1709, 1607, 1508, 1452, 1390, 1366, 1271, 1249, 1145, 1099, 981, 886, 852, 811, 779, 512, 496. HRMS (ESI): *m/z* calcd for [M+H]^+^, C_52_H_91_N_12_O_17_, 1155.6612; found, 1155.6620. Anal. Calcd. for C_52_H_90_N_12_O_17_·0.5H_2_O: C, 53.64; H, 7.88; N, 14.44; found: C, 53.69; H, 8.11; N, 14.21. [α]^25^_D_ = +22.3 (*c* = 1.0, CHCl_3_).

5,5-Bis[3-(l-alanyl)aminopropyl]barbituric acid (**11i**)

Compound **11d** (20 mg, 0.03 mmol) was dissolved in MeOH (0.5 mL), to which conc. HCl (0.5 mL) was added and the reaction mixture was stirred at room temperature for 12 h. The resulting solution was evaporated under reduced pressure to give **11i** as a white solid (16 mg, quant.). ^1^H NMR (300 MHz, CD_3_OD, TMS): *δ* = 3.88 (q, *J* = 7.2 Hz, 2H), 3.26–3.10 (m, 4H), 1.96–1.91 (m, 4H), 1.49 (d, *J* = 7.2 Hz, 6H), 1.46–1.39 (m, 4H) ppm; ^13^C NMR (100 MHz, CD_3_OD, TMS): *δ* = 174.6, 170.8, 56.5, 50.3, 40.1, 37.1, 26.1, 18.4, 17.7 ppm; mp 194 °C; IR (ATR): *ν* = 2933, 1749, 1722, 1666, 1563, 1494, 1447, 1355, 1268, 1206, 1115, 1043, 829, 760, 667, 496. HRMS (ESI): *m/z* calcd for [M+H]^+^, C_16_H_29_N_6_O_5_, 385.2195; found, 385.2194. Anal. Calcd. for C_16_H_28_N_6_O_5_·2HCl·H_2_O·1.5MeOH: C, 40.16; H, 7.32; N, 16.06; found: C, 40.13; H, 7.01; N, 16.09. [α]^25^_D_ = +2.24 (*c* = 0.5, CH_3_OH).

5,5-Bis[3-(l-seryl)aminopropyl]barbituric acid (**11j**)

Compound **11e** (10 mg, 0.01 mmol) was dissolved in MeOH (0.3 mL), to which conc. HCl (0.3 mL) was added and the reaction mixture was stirred at room temperature for 12 h. The resulting solution was evaporated under reduced pressure to give **11j** as a white solid (7 mg, quant.). ^1^H NMR (400 MHz, CD_3_OD, TMS): *δ* = 3.95–3.90 (m, 4H), 3.80 (q, *J* = 7.2 Hz, 2H), 3.19 (t, *J* = 7.2 Hz, 4H), 1.96–1.92 (m, 4H), 1.47–1.40 (m, 4H) ppm; ^13^C NMR (100 MHz, CD_3_OD, TMS): *δ* = 173.2, 166.8, 149.7, 60.4, 55.0, 38.8, 35.6, 33.5, 24.6 ppm; mp 197 °C; IR (ATR): *ν* = 3197, 3059, 2932, 1748, 1667, 1562, 1495, 1448, 1418, 1356, 1338, 1308, 1272, 1210, 1145, 1045, 761, 498. HRMS (ESI): *m/z* calcd for [M+H]^+^, C_16_H_29_N_6_O_7_, 417.2090; found, 417.2092. Anal. Calcd. for C_16_H_28_N_6_O_7_·2HCl·1.5H_2_O·1.5MeOH: C, 37.24; H, 6.96; N, 14.89; found: C, 36.96; H, 6.44; N, 14.72. [α]^25^_D_ = +5.90 (*c* = 0.5, CH_3_OH).

5,5-Bis[3-(l-phenylalanyl)aminopropyl]barbituric acid (**11k**)

Compound **11f** (25 mg, 0.03 mmol) was dissolved in MeOH (0.5 mL), to which conc. HCl (0.5 mL) was added and the reaction mixture was stirred at room temperature for 12 h. The resulting solution was evaporated under reduced pressure to give **11k** as a white solid (22 mg, quant.). ^1^H NMR (300 MHz, CD_3_OD, TMS): *δ* = 7.35–7.24 (m, 10H), 3.98 (t, *J* = 7.2 Hz, 2H), 3.19–3.13 (m, 2H), 3.10 (d, *J* = 7.2 Hz, 4H), 2.97–2.90 (m, 2H), 1.81 (t, *J* = 8.4 Hz, 4H), 1.23–1.15 (m, 4H) ppm; ^13^C NMR (100 MHz, CD_3_OD, TMS): *δ* = 174.5, 169.3, 151.2, 135.7, 130.5, 130.1, 128.9, 56.3, 55.9, 40.0, 38.8, 37.1, 25.9 ppm; mp 183–185 °C; IR (ATR): *ν* = 3189, 3029, 2933, 2818, 1749, 1721, 1668, 1563, 1495, 1454, 1355, 1335, 1303, 1268, 1207, 1144, 1080, 1041, 825, 746, 700, 497. HRMS (ESI): *m/z* calcd for [M+H]^+^, C_28_H_37_N_6_O_5_, 537.2820; found, 537.2820. Anal. Calcd. for C_28_H_36_N_6_O_5_·2HCl·1.5H_2_O·MeOH: C, 52.10; H, 6.78; N, 12.57; found: C, 52.17; H, 6.59; N, 12.64. [α]^25^_D_ = +34.9 (*c* = 0.5, CH_3_OH).

5,5-Bis[3-(l-tyrosyl)aminopropyl]barbituric acid (**11l**)

A solution of **11g** (25 mg, 0.03 mmol) and conc. HCl (0.5 mL) in MeOH (0.5 mL) was stirred at room temperature for 12 h. The resulting solution was evaporated under reduced pressure to give **11l** as a white solid (21 mg, quant.). ^1^H NMR (300 MHz, CD_3_OD, TMS): *δ* = 7.04 (d, *J* = 8.7 Hz, 4H), 6.78 (d, *J* = 8.7 Hz, 4H), 3.91 (t, *J* = 7.2 Hz, 2H), 3.16–2.90 (m, 8H), 1.87–1.82 (m, 4H), 1.37–1.31 (m, 4H) ppm; ^13^C NMR (100 MHz, CD_3_OD, TMS): *δ* = 174.5, 169.7, 158.2, 151.1, 131.6, 126.2, 116.9, 56.5, 56.1, 40.2, 38.0, 37.1, 25.8 ppm; mp 196–198 °C; IR (ATR): *ν* = 3019, 2934, 1749, 1722, 1668, 1613, 1564, 1514, 1443, 1355, 1306, 1210, 1041, 824, 780, 632, 497. HRMS (ESI): *m/z* calcd for [M+H]^+^, C_28_H_37_N_6_O_7_, 569.2707; found, 569.2718. Anal. Calcd. for C_28_H_36_N_6_O_7_·4HCl·H_2_O·MeOH: C, 45.56; H, 6.06; N, 10.99; found: C, 45.42; H, 5.87; N, 11.13. [α]^25^_D_ = +35.6 (*c* = 1.0, CH_3_OH).

5,5-Bis[3-(l-arginyl)aminopropyl]barbituric acid (**11m**)

A solution of **11h** (40 mg, 0.03 mmol) and conc. HCl (0.5 mL) in MeOH (0.5 mL) was stirred at room temperature for 12 h. The resulting solution was evaporated under reduced pressure to give **11m** as a white solid (22 mg, quant.). ^1^H NMR (300 MHz, CD_3_OD, TMS): *δ* = 3.89 (t, *J* = 6.0 Hz, 2H), 3.30–3.26 (m, 8H), 3.18–3.09 (m, 2H), 1.98–1.87 (m, 8H), 1.74–1.67 (m, 4H), 1.45–1.43 (m, 4H) ppm; ^13^C NMR (100 MHz, CD_3_OD, TMS): *δ* = 174.6, 169.9, 158.7, 151.0, 56.5, 54.1, 41.9, 40.2, 37.2, 29.8, 26.0, 25.6 ppm; mp 182–184 °C; IR (ATR): *ν* = 3157, 3058, 2936, 1749, 1722, 1655, 1561, 1498, 1446, 1355, 1303, 1270, 1209, 1162, 1042, 827, 663, 496. HRMS (ESI): *m/z* calcd for [M+H]^+^, C_22_H_43_N_12_O_5_, 555.3480; found, 555.3474. Anal. Calcd. for C_22_H_42_N_12_O_5_·4HCl·2H_2_O·MeOH: C, 35.94; H, 7.08; N, 21.87; found: C, 35.87; H, 6.89; N, 21.83. [α]^25^_D_ = +10.7 (*c* = 1.0, CH_3_OH).

5,5-Bis{3-[*N*-(9-fluorenylmethoxycarbonyl)-*O*-(*t*-butyl)-l-seryl]aminopropyl}barbituric acid (**11n**)

Compound **11n** was synthesized as a white solid (31 mg, 98% yield) from **11c** (10 mg, 0.03 mmol), Fmoc-l-Serine(OtBu)-OH (27 mg, 0.07 mmol), diisopropylethylamine (22 mg, 0.17 mmol), and PyBOP (36 mg, 0.07 mmol) in anhydrous DMF (0.5 mL) using a procedure similar to that used for **11d**. ^1^H NMR (300 MHz, CDCl_3_, TMS): *δ* = 9.03 (brs, 2H), 7.75 (d, *J* = 7.2 Hz, 4H), 7.62–7.54 (m, 4H), 7.39 (t, *J* = 7.2 Hz, 4H), 7.29 (t, *J* = 7.2 Hz, 4H), 6.70–6.58 (m, 2H), 5.80 (d, *J* = 7.2 Hz, 2H), 4.40 (d, *J* = 6.9 Hz, 4H), 4.21 (t, *J* = 7.2 Hz, 4H), 3.76 (dd, *J* = 3.9 Hz, 2H), 3.38 (t, *J* = 8.1 Hz, 2H), 3.28–3.10 (m, 4H), 2.02–1.88 (m, 4H), 1.50–1.36 (m, 4H), 1.17 (s, 18H) ppm; ^13^C NMR (100 MHz, CDCl_3_, TMS): *δ* = 172.0, 170.5, 156.2, 148.6, 143.8, 141.3, 127.7, 127.1, 125.2, 120.0, 74.2, 67.1, 61.7, 55.6, 54.6, 47.1, 39.0, 35.7, 27.4, 24.9 ppm; mp 122–124 °C; IR (ATR): *ν* = 3313, 3064, 2972, 2934, 2870, 1698, 1659, 1516, 1449, 1410, 1389, 1363, 1330, 1235, 1191, 1081, 1023, 874, 758, 738, 664, 620, 539, 496. HRMS (ESI): *m/z* calcd for [M+H]^+^, C_54_H_65_N_6_O_11_, 973.4707; found, 973.4706. Anal. Calcd. for C_54_H_64_N_6_O_11_·H_2_O: C, 65.44; H, 6.71; N, 8.48; found: C, 65.23; H, 6.77; N, 8.30. [α]^25^_D_ = +20.4 (*c* = 1.0, CHCl_3_).

5,5-Bis{3-[*O*-(*t*-butyl)-l-seryl]aminopropyl}barbituric acid (**11o**)

To a solution of **11n** (15 mg, 0.02 mmol) in anhydrous DMF (0.4 mL), piperidine (0.1 mL) was added and the reaction mixture was stirred at room temperature for 20 min. The reaction mixture was concentrated under reduced pressure, and resulting residue was purified by silica gel column chromatography (AcOEt/MeOH = 1/0 to 6/1) to give **11o** as a colorless amorphous solid (7 mg, 92% yield). ^1^H NMR (400 MHz, CDCl_3_, TMS): *δ* = 7.53–7.48 (m, 2H), 3.62 (q, *J* = 4.0 Hz, 2H), 3.53 (q, *J* = 4.0 Hz, 2H), 3.44 (t, *J* = 8.0 Hz, 2H), 3.24–3.14 (m, 4H), 1.98–1.90 (m, 4H), 1.50–1.36 (m, 4H), 1.18 (s, 18H) ppm; ^13^C NMR (100 MHz, CDCl_3_, TMS): *δ* = 173.2, 173.0, 150.2, 73.6, 64.0, 55.6, 55.5, 38.8, 35.9, 27.6, 25.1 ppm; IR (ATR): *ν* = 3299, 3072, 2971, 2928, 2856, 1723, 1693, 1646, 1533, 1448, 1411, 1388, 1362, 1335, 1306, 1258, 1192, 1078, 1022, 874, 759, 670, 493. HRMS (ESI): *m/z* calcd for [M+H]^+^, C_24_H_45_N_6_O_7_, 529.3344; found, 529.3344. [α]^25^_D_ = −14.5 (*c* = 1.4, CHCl_3_).

5,5-Bis{3-[*N*-(9-fluorenylmethoxycarbonyl)-l-seryl]aminopropyl}barbituric acid (**11p**)

To a solution of **11n** (11 mg, 0.01 mmol) in dichloromethane (0.2 mL), trifluoroacetic acid (0.2 mL) was added and the reaction mixture was stirred at room temperature for 2 h. The reaction mixture was concentrated under reduced pressure, suspended in H_2_O, extracted with AcOEt (10 mL × 3), washed with brine, dried over Na_2_SO_4_, and concentrated under reduced pressure. The resulting residue was purified by silica gel column chromatography (AcOEt/Hexanes = 4/1 to 1/0) to give **11p** as a white solid (5 mg, 54% yield). ^1^H NMR (400 MHz, CD_3_OD, TMS): *δ* = 7.78 (d, *J* = 7.6 Hz, 4H), 7.67 (t, *J* = 7.6 Hz, 4H), 7.38 (t, *J* = 7.6 Hz, 4H), 7.30 (t, *J* = 7.6 Hz, 4H), 4.39 (dd, *J* = 2.4 Hz, 4H), 4.23 (t, *J* = 6.8 Hz, 2H), 4.12 (t, *J* = 6.8 Hz, 2H), 3.72 (d, *J* = 5.2 Hz, 4H), 3.17–3.08 (m, 4H), 1.96–1.84 (m, 4H), 1.43–1.34 (m, 4H) ppm; ^13^C NMR (100 MHz, CD_3_OD, TMS): *δ* = 174.6, 172.9, 158.5, 151.0, 145.4, 145.3, 142.6, 128.8, 128.2, 126.3, 121.0, 68.1, 63.3, 58.7, 56.5, 40.0, 36.9, 25.9 ppm; mp 124–125 °C; IR (ATR): *ν* = 3316, 3067, 2932, 2854, 1695, 1657, 1528, 1448, 1415, 1334, 1245, 1203, 1137, 1060, 758, 738, 620, 538, 498. HRMS (ESI): *m/z* calcd for [M+Na]^+^, C_46_H_48_N_6_O_11_Na, 883.3283; found, 883.3273. Anal. Calcd. for C_46_H_48_N_6_O_11_·2H_2_O: C, 61.60; H, 5.84; N, 9.37; found: C, 61.77; H, 5.69; N, 8.98. [α]^25^_D_ = −9.50 (*c* = 0.7, CH_3_OH).

### 2.3. Hydrolysis of MNP

The hydrolysis of MNP was carried out in 10 mM HEPES buffer (pH 7.4) with *I* = 0.1 (NaNO_3_) (for **8, 15**) and in a CHCl_3_/50 mM HEPES buffer (pH 7.4) with *I* = 0.1 (NaNO_3_) (2/8) (for **9**, **10**, **16**, **17**) at 37 °C. Stock solutions of **1** (3.0 mM in H_2_O), **4** or **11** (6.0 mM in H_2_O), and CuNO_3_·3H_2_O (10 mM in H_2_O) were used for the preparation of sample solutions of **8** and **15** (20 *μ*M) in 10 mM HEPES buffer (pH 7.4) with *I* = 0.1 (NaNO_3_) (3.0 mL). Stock solutions of **2** (1.0 mM in CHCl_3_), **4** or **11** (3.0 mM in H_2_O or CHCl_3_), and Cu(ClO_4_)_2_·6H_2_O (20 mM in H_2_O) were used for the preparation of sample solutions of **9** and **16** (20 *μ*M in total solution) in CHCl_3_/50 mM HEPES buffer (pH 7.4) with *I* = 0.1 (NaNO_3_) (2/8) (total volume 3.0 mL). A stock aqueous solution of MNP (20 mM) was used for the hydrolysis reaction. Prior to the hydrolysis of MNP in the two-phase solvent system, the reaction mixtures of **2** or **3**, barbital derivatives (**4a**–**c**, **11a**–**p**), and Cu^2+^ in CHCl_3_/50 mM HEPES buffer (pH 7.4) with *I* = 0.1 (NaNO_3_) (2/8) were incubated at 37 °C overnight using a shaking water bath (shaking speed: 150 rpm) (Tokyo Glass Kikai, FWB-1) to form **9**, **10**, **16**, and **17** *in situ*, and the aqueous solution of MNP was added. All of the hydrolysis experiments were performed in triplicate at 37 °C using a shaking water bath (shaking speed: 150 rpm). The yields of the hydrolysis product from MNP in the presence of the supramolecular complexes were calculated based on the increase in the absorption of the released 4-nitrophenol (NP) at 400 nm ([NP produced in the presence of the supramolecular complex]–[NP produced in the absence of the supramolecular complex]) (*ε*_400_ value of NP is 1.35 × 10^4^ M^−1^·cm^−1^ at pH 7.4) [17] in aqueous layer. The partition ratio of NP (79% in aqueous solution), which had been determined in the previous report [53], was used for the calculation of the yields in the CHCl_3_/H_2_O system. 

## 3. Results and Discussion

### 3.1. Synthesis of Barbital Derivatives

**Scheme 4:**
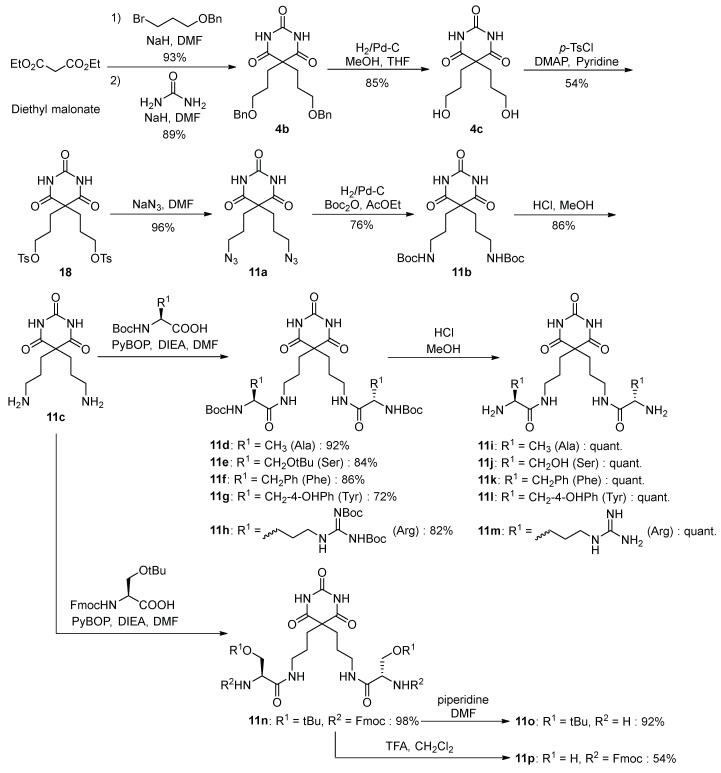
Synthesis of barbital derivatives **11a**–**p** as building units for supramolecular complexes **15**, **16** and **17**.

The barbital derivatives that were functionalized with amino acid residues were synthesized as shown in Scheme 4. Compound **4c** was synthesized from diethyl malonate via **4b** according to our previous paper [24] and was reacted with *p*-toluenesulfonyl chrolide to afford the ditosylate **18**. The reaction of **18** with sodium azide gave compound **11a**. Our attempt to carry out Staudinger reactions [55,56] of **11a** with the *o*-phosphynothioesters of amino acids resulted in failure. Therefore, the reduction of the azide groups of **11a** were carried out to obtain the diamino intermediate **11c**. It should be noted that two amino groups of **11c** produced by the reduction of **11a** were directly protected with a Boc group *in situ* for easy purification as a Boc-protected compound **11b** and the subsequent deprotection of the Boc groups afforded **11c**. The condensation reactions of **11c** with Boc-protected amino acids in DMF in the presence of PyBOP and DIEA gave **11d**–**h**. The deprotection of the Boc groups by treatment with HCl afforded the corresponding compounds **11i**–**m** in quantitative yield. The barbital derivative equipped with serine **11n** was obtained from **11c** and Fmoc-l-Ser(OtBu)-OH and then reacted with piperidine and TFA to give compound **11o** and **11p**, respectively.

### 3.2. Complexation Behavior of 1 (Zn_2_L^1^) with Barbital Derivatives and Cu^2+^ by UV/Vis Titrations

**Figure 1:**
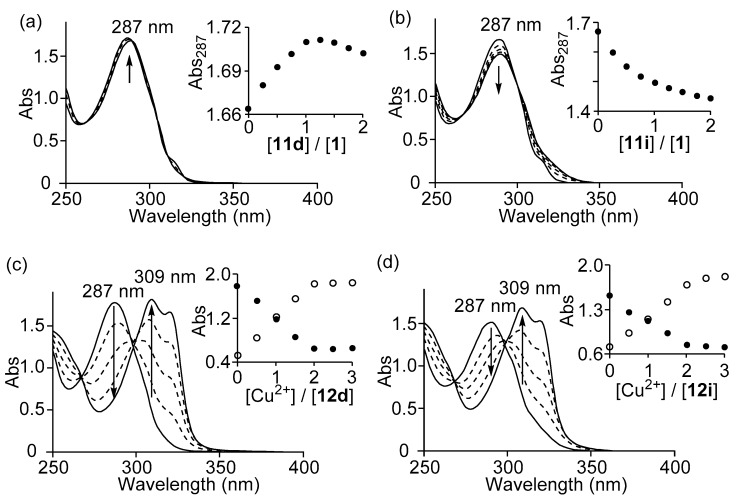
(**a**) UV/Vis titration of **1** (80 *μ*M) upon the addition of **11d** in 10 mM HEPES buffer (pH 7.4 with *I* = 0.1 (NaNO_3_)) at 25 °C; (**b**) UV/Vis titration of **1** (80 *μ*M) upon the addition of **11i** in 10 mM HEPES buffer (pH 7.4 with *I* = 0.1 (NaNO_3_)) at 25 °C; (**c**) UV/Vis titration of **12d** (40 *μ*M) upon the addition of Cu^2+^ in 10 mM HEPES buffer (pH 7.4 with *I* = 0.1 (NaNO_3_)) at 25 °C; (**d**) UV/Vis titration of **12i** (40 *μ*M) upon the addition of Cu^2+^ in 10 mM HEPES buffer (pH 7.4 with *I* = 0.1 (NaNO_3_)) at 25 °C. The insets in (**a**,**b**) show the change in the absorbance at 287 nm, and the insets in (**c**,**d**) show the decrease in absorbance at 287 nm (closed circles) and the increase in absorbance at 309 nm (open circles).

In order to examine the formation of the 2:2 and 2:2:2 supramolecular complexes from the bis(Zn^2+^-cyclen) complexes **1** (Zn_2_L^1^) and **2** (Zn_2_L^2^) with the synthesized Bar units and Cu^2+^ as shown in Scheme 1, UV/Vis titrations of the bis(Zn^2+^-cyclen) complexes with **11d** and **11i**, and then with Cu(NO_3_)_2_·3H_2_O were attempted. While the titrations of **2** in the two-phase solvent system (CHCl_3_/50 mM HEPES buffer (pH 7.4, *I* = 0.1 (NaNO_3_)) with **11d** and **11i** and Cu(ClO_4_)_2_·6H_2_O were unsuccessful, the UV/Vis titrations of **1** with **11d** and **11i** were successfully carried out in 10 mM HEPES buffer (pH 7.4, *I* = 0.1 (NaNO_3_)) at 37 °C. As shown in Figure 1a,b, 80 *μ*M of **1** has an absorption maxima (*λ*_max_) at 287 nm, which increased upon the addition of **11d** and decreased upon the addition of **11i**, reaching a plateau at a 1:1 ratio, suggesting a 2:2 assembly of **1** with **11d** or **11i** as same as that with **4a**. In addition, as shown in Figure 1c,d, the addition of Cu^2+^ induced a red shift from 287 nm to 309 nm, which reached a plateau at [**12d**] (or [**12i**]):[Cu^2+^] = 1:2, suggesting the quantitative formation of the 2:2:2 supermolecules **15** with newly synthesized barbital derivatives at *μ*M order concentrations.

### 3.3. Location of Complexes 13 and 16 in the Two-Phase Solvent System, as Determined by UV/Vis Spectra

**Figure 2:**
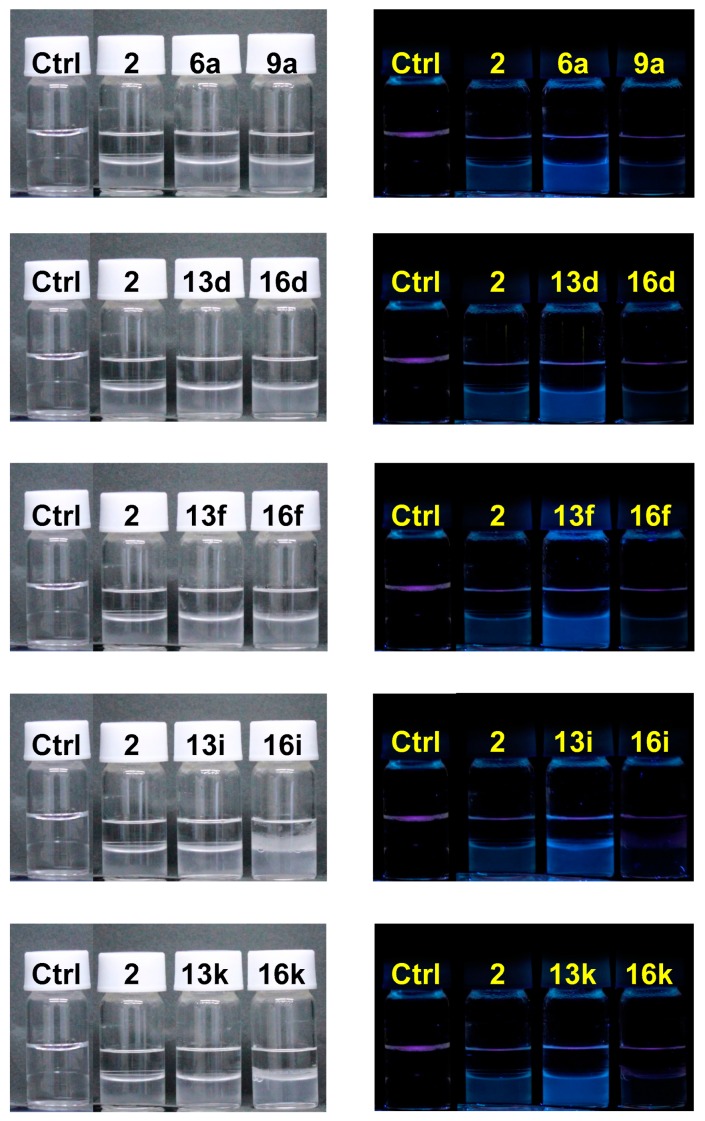
Photos of mixtures of **2** (Zn_2_L^2^), the complexes of **2** with barbital derivatives (**4a**, **11d**, **11f**, **11i**, and **11k**) (**6a**, **13d**, **13f**, **13i**, and **13k**), and the 2:2:2 complexes of **2**, Bar units and Cu^2+^ (**9a**, **16d**, **16f**, **16i**, and **16k**) in CHCl_3_/50 mM HEPES buffer (pH 7.4) with *I* = 0.1 (NaNO_3_) (1/1) at room temperature.

The distribution of **13** and **16** in the organic phase and the aqueous phase of the two-phase solvent system was determined from the UV/Vis absorption spectra of both layers, as described in our previous report [54]. For the *in situ* formation of **13** and **16**, **11d** and **11f** were selected as more hydrophobic barbital units, and **4a**, **11i,** and **11k** were selected as more hydrophilic barbital units. Two-phase solutions of Zn_2_L^2^ (**2**) alone (40 *μ*M), 2:2 complex (20 *μ*M) of Zn_2_L^2^ and barbital derivatives (**6a**, **13d**, **13f**, **13i**, and **13k**), and 2:2:2 complex (20 *μ*M) of Zn_2_L^2^, barbital derivatives, and Cu^2+^ (**9a**, **16d**, **16f**, **16i**, and **16k**) were prepared in CHCl_3_/50 mM HEPES buffer (pH 7.4) with *I* = 0.1 (NaNO_3_) (1/1), incubated for 18 h at 37 °C, and centrifuged (2000 rpm × 10 min) at room temperature. Pictures of each complex and their distribution ratios are summarized in Figure 2 and Table 1. The findings indicate that **2** is located mostly in the organic layer (*ca.* 99%), and the addition of barbital derivatives did not significantly affect the distribution of **6a**, **13d**, **13f**, **13i**, and **13k** (*ca.* 96%–>99%), implying that these complexes are distributed mainly in the organic layer. Since the addition of Cu^2+^ quenched the emission from the bpy units, it was not possible to determine the distribution of **9a**, **16d**, **16f**, **16i**, and **16k** in both layers. These behaviors of **6a**, **13d**, **13f**, **13i**, and **13k** are different from **7a** prepared from **3** (Zn_2_L^3^) and **4a** (Bar), which were distributed both in the aqueous and the organic layers [54].

### 3.4. Hydrolysis of MNP by 2:2:2 Complexes in a Two-Phase Solvent System

**Figure 3:**
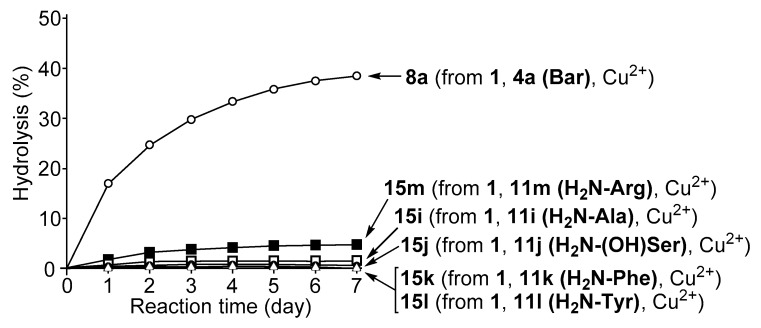
Hydrolysis of MNP (100 *μ*M in the total solution) by **8a** (open circles), **15i** (open squares), **15j** (closed circles), **15k** (open triangles), **15l** (open triangles), or **15m** (closed squares) ([**8** or **15**] = 100 *μ*M) in a single aqueous phase (10 mM HEPES buffer (pH 7.4) with *I* = 0.1 (NaNO_3_)) at 37 °C.

We conducted the hydrolysis of MNP (100 *μ*M) in the single-phase solvent system (10 mM HEPES buffer (pH 7.4) with *I* = 0.1 (NaNO_3_)) at 37 °C in the presence of **8a** or **15i**–**m** (prepared from **1**, **11i**–**m**, and Cu^2+^) (100 *μ*M) [24] and in the two-phase solvent system (CHCl_3_/50 mM HEPES buffer (pH 7.4) with *I* = 0.1 (NaNO_3_)) (2/8) at 37 °C in the presence of **9a** or **16i**–**m** (prepared from **2**, **11i**–**m**, and Cu^2+^) (20 *μ*M in the total solution including the aqueous phase and the CHCl_3_ phase). The results showed that the MNP hydrolysis in the presence of **15i**–**m** was much slower than that of **8a** in the single-phase solvent system, as shown in Figure 3.

**Figure 4:**
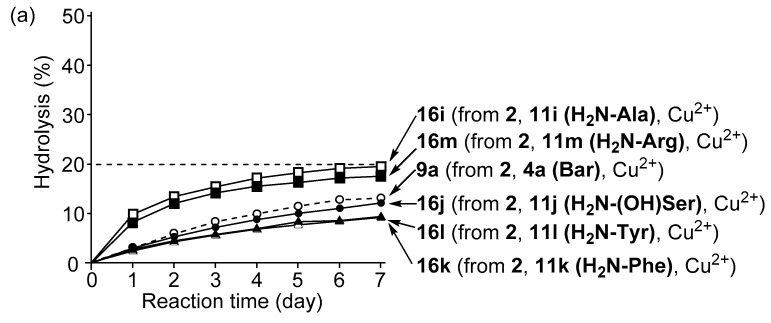

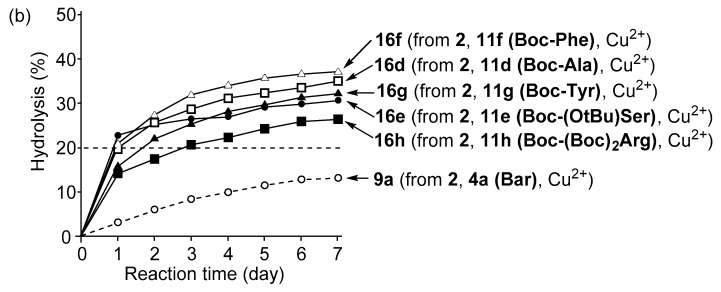
Hydrolysis of MNP (100 *μ*M in the total solution) by (**a**) **9a** (open circles with dashed curve), **16i** (open squares), **16j** (closed circles), **16k** (open triangles), **16l** (closed triangles), or **16m** (closed squares) ([**9**, **16**] = 20 *μ*M in the total solution); (**b**) **9a** (open circles with dashed curve), **16d** (open squares), **16e** (closed circles), **16f** (open triangles), **16g** (closed triangles), or **16h** (closed squares) ([**9**, **16**] = 20 *μ*M in the total solution) in CHCl_3_/50 mM HEPES buffer (pH 7.4) with *I* = 0.1 (NaNO_3_) (2/8) at 37 °C. The concentrations of the product (4-nitrophenol) were determined in the total two-phase solvent system.

**Figure 5:**
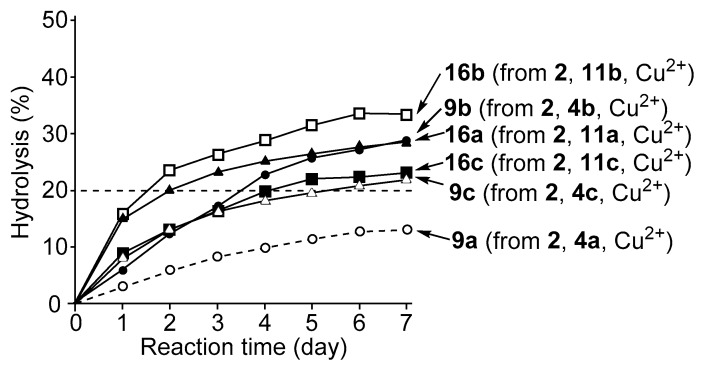
Hydrolysis of MNP (100 *μ*M in the total solution) by **9a** (open circles), **9b** (closed triangles), **9c** (open triangles), **16a** (closed circles), **16b** (open squares), and **16c** (closed squares) ([**9**, **16**] = 20 *μ*M in the total solution) in CHCl_3_/50 mM HEPES buffer (pH 7.4) with *I* = 0.1 (NaNO_3_) (2/8) at 37 °C. The concentrations of the product (4-nitrophenol) were determined in the total two-phase solvent system.

As shown in Figure 4a, the activity of **16i**–**m** (20 *μ*M) was similar to that of **9a** in the two-phase solvent system with negligible catalytic turnover (note that [**16i**–**m**] = 20 *μ*M), suggesting that more hydrophobic supermolecules have higher hydrolysis activity in the two-phase solvent system than in the single-phase solvent system. Interestingly, the MNP hydrolysis activity for **16d**–**h** (prepared from **2**, **11d**–**h**, and Cu^2+^) was higher than that of **16i**–**m** under the same conditions, as shown in Figure 4b, and the hydrolysis yields for **16d** and **16f** were in excess of 20% after 1 day and in excess of 25%–40% after 7 days, indicating that these complexes had catalytic activities. Moreover, the activities of **16a** and **16b** were similar to that for **16d**–**h** (Figure 5), suggesting that the azide and Boc-protected amino groups on the Bar unit provide a similar effect on the hydrolysis of MNP as those of the protected amino acid residues in **16d**–**g**.

**Figure 6:**
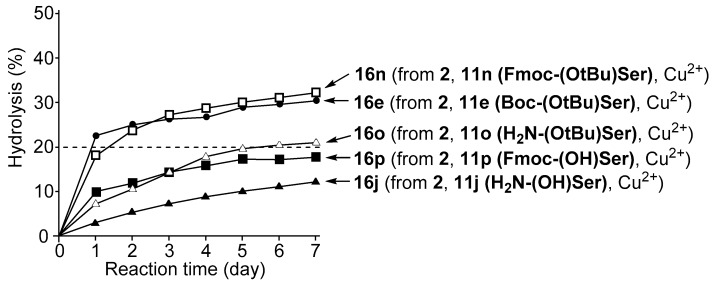
Hydrolysis of MNP (100 *μ*M in the total solution) by **16e** (closed circles), **16j** (closed triangles), **16n** (open squares), **16o** (open triangles), and **16p** (closed squares) (barbital derivatives containing serine residue) ([**16**] = 20 *μ*M in the total solution) in CHCl_3_/50 mM HEPES buffer (pH 7.4) with *I* = 0.1 (NaNO_3_) (2/8) at 37 °C. The concentrations of the product (4-nitrophenol) were determined in the total solution of the two-phase solvent system.

Figure 6 summarizes the results for the hydrolysis of MNP by **16e**, **16j**, **16n**, **16o**, and **16p**, which contain serine units ([MNP] = 100 *μ*M and [**16e**, **16j**, **16n**, **16o**, and **16p**] = 20 *μ*M). The findings suggest that **16e** and **16n** consisting of the fully protected Ser have a higher activity than **16j**, **16o**, and **16p** in which the Bar units contain fully or partially deprotected Ser.

**Figure 7:**
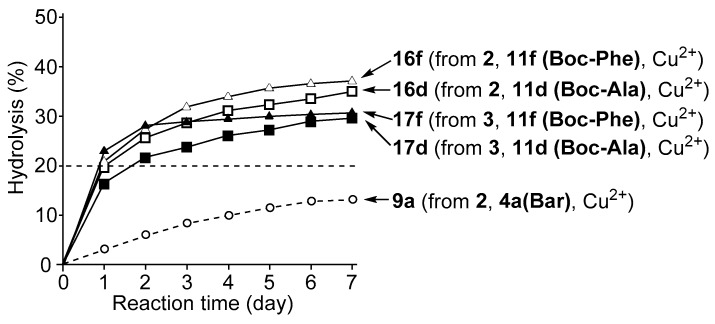
Hydrolysis of MNP (100 *μ*M in the total solution) by **9a** (open circles), **16d** (open squares), **16f** (open triangles), **17d** (closed squares), and **17f** (closed triangles) ([**9**, **16**, **17**] = 20 *μ*M in the total solution) in CHCl_3_/50 mM HEPES buffer (pH 7.4) with *I* = 0.1 (NaNO_3_) (2/8) at 37 °C. The concentrations of the product (4-nitrophenol) were determined in the total two-phase solvent system.

The hydrolysis of MNP (100 *μ*M) by the amphiphilic supramolecular complexes **17d** or **17f** (prepared from **3**, **11d,** or **11f**, and Cu^2+^) (20 *μ*M) was also examined. As shown in Figure 7, the hydrolytic activity of **17d** and **17f** were higher than **9a**, but slightly lower than **16d** and **16f**.

**Figure 8:**
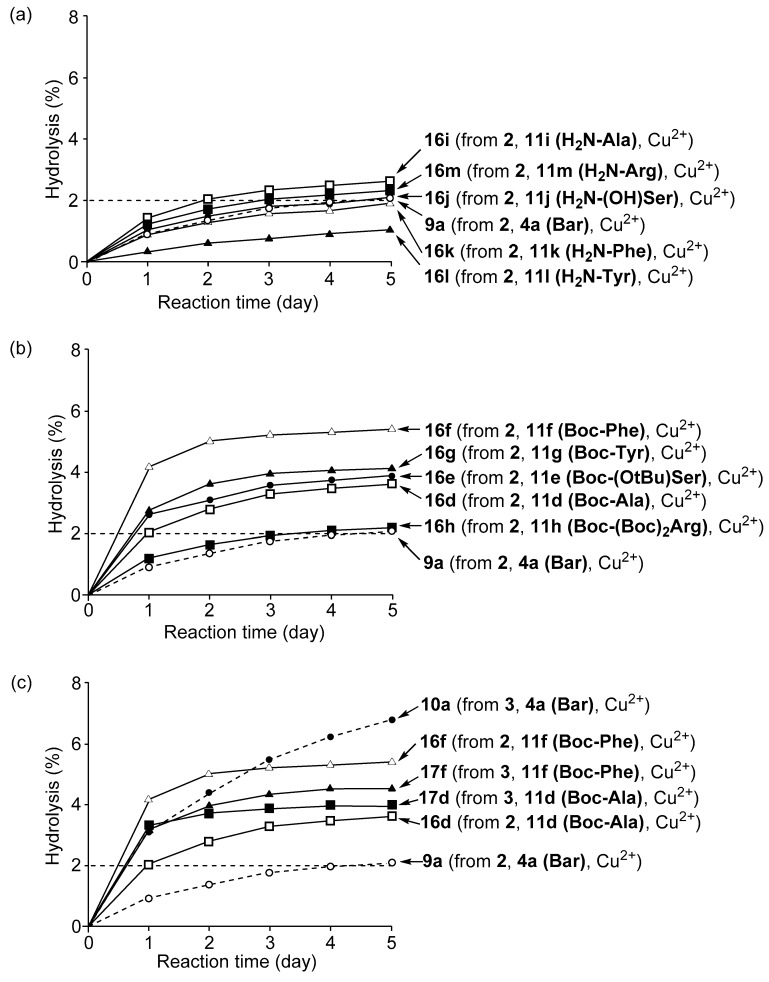
Hydrolysis of MNP (1000 *μ*M in the total solution) by (**a**) **9a** (open circles with dashed curve), **16i** (open squares), **16j** (closed circles), **16k** (open triangles), **16l** (closed triangles), or **16m** (closed squares); (**b**) The hydrolysis of MNP by **9a** (open circles with dashed curve), **16d** (open squares), **16e** (closed circles), **16f** (open triangles), **16g** (closed triangles), or **16h** (closed squares); (**c**) The hydrolysis of MNP by **9a** (open circles with dashed curve), **10a** (closed circles with dashed curve), **16d** (open squares), **16f** (open triangles), **17d** (closed squares), **17f** (closed triangles) ([**9**, **16**, **17**] = 20 *μ*M in the total solution) in CHCl_3_/50 mM HEPES buffer (pH 7.4) with *I* = 0.1 (NaNO_3_) (2/8) at 37 °C. The concentrations of the product (4-nitrophenol) were determined in the total two-phase system.

**Figure 9:**
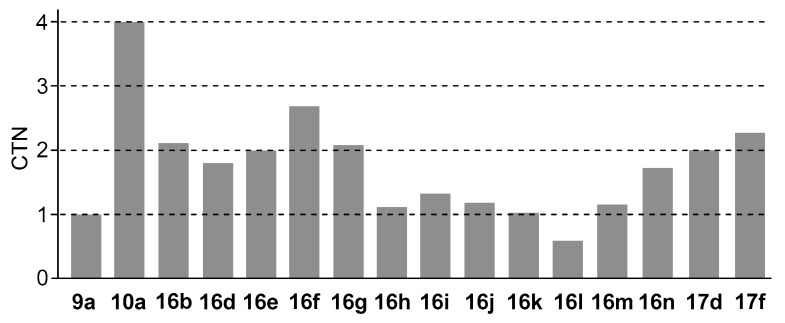
Comparison of catalytic turnover numbers (CTN) of **9a**, **10a**, **16b**, **16d**–**n**, **17d**, and **17f** at [MNP] = 1000 *μ*M and [**9**, **16**, **17**] = 20 *μ*M (in the total solution) in CHCl_3_/50 mM HEPES buffer (pH 7.4) with *I* = 0.1 (NaNO_3_) (2/8) at 37 °C.

The hydrolysis of MNP by **16d**–**m**, **17d** and **17f** (20 *μ*M) was carried out at higher concentrations of MNP (200, 300, 400, 500, and 1000 *μ*M) and the typical results at [MNP] = 1000 *μ*M are summarized in Figure 8 (the results for **9a** are included in all of the graphs as standard data), in which **16d**–**g** formed from the *N*-protected barbital units **11d**–**g** showed higher hydrolysis activities. It should be noted that **16f** demonstrated the highest MNP hydrolysis yield as shown in Figure 8b, and the catalytic turnover number (CTN) was more than 2 after 1 day. The CTN values for **9a**, **10a**, **16b**, **16d**–**n**, **17d**, and **17f** at [MNP] =1000 *μ*M, which are summarized in Figure 9, imply that the CTNs of **16b**, **16e**, **16f**, **16g**, **17d**, and **17f** are over 2, while these values are smaller than that of our previously reported **10a** (~4) [54].

### 3.5. Michaelis–Menten Kinetics for Hydrolysis of MNP by 9, 16, and 17 in the Two-Phase Solvent System

**Figure 10:**
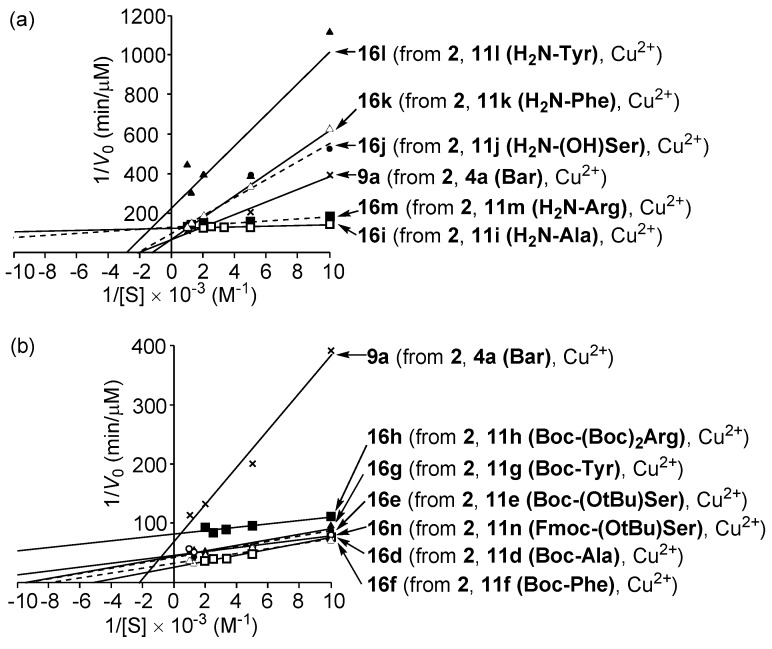
(**a**) Lineweaver–Burk plots for the hydrolysis of MNP catalyzed by **9a** (cross with plain line), **16i** (open squares with plain line), **16j** (closed circles with dashed line), **16k** (open triangles with plain line), **16l** (closed triangles with plain line), and **16m** (closed squares with dashed line). (**b**) Lineweaver–Burk plots for the hydrolysis of MNP by **9a** (crosses with plain line), **16d** (open squares with plain line), **16e** (closed circles with dashed line), **16f** (open triangles with dashed line), **16g** (closed triangles with plain line), **16h** (closed squares with plain line), and **16n** (open circles with plain line).

The results for the hydrolysis of MNP by the supramolecular phosphatases at [MNP] = 100, 200, 500, 800, and 1000 *μ*M (in the total solution) were analyzed based on Michaelis–Menten kinetics, as described in our previous reports (the hydrolysis of MNP by **16d**, **16h**, and **16i** were carried out at [MNP] = 100, 200, 300, 400, and 500 *μ*M (in the total solution)) [19,24,53,54]. Based on the Lineweaver–Burk plots shown in Figure 10, *V*_max_ (the maximum velocity for the formation of NP from MNP promoted by **9**, **16**, and **17**, *μ*M min^−1^), *K*_m_ (Michaelis constant, *μ*M), and *k* (the first-order rate constant defined by Equation (1), min^−1^) [57] values were determined and summarized in Table 2.

*k* = *V*_max_/*K*_m_ (1)

The *V*_max_ and *K*_m_ values for **9a** were reported to be (1.4 ± 0.4) × 10^−2^ *μ*M min^−1^ and (5.4 ± 0.5) × 10^2^ *μ*M (entry 3 in Table 2), respectively, in a two-phase solvent system in our previous report [53]. Among **16b**, **16d**–**n**, **17d,** and **17f** tested in this work (entry 5–18 in Table 2), **16d** had the highest *V*_max_ (3.9 × 10^−2^ *μ*M min^−1^) (entry 6) and **16f** had the highest CTN (2.7) (entry 8).

The hydrolysis of MNP by **9a**, **16d**, **16f**, **16i**, **17d**, and **17f** in the presence of HPO_4_^2−^, a product and an inhibitor of MNP hydrolysis, was also carried out to determine the *K*_i_ values of HPO_4_^2−^ as listed in Table 2. Interestingly, it was indicated that **9a**, **16d**, **16f**, and **16i** are inhibited by inorganic phosphate in a competitive manner and the *K*_i_ values for **16d** (16 *μ*M) and **16f** (23 *μ*M) (entries 6 and 8) was almost comparable to that for **9a** (*ca.* 15 *μ*M) (entry 3). In contrast, the *K*_i_ value for **16i** is 0.67 *μ*M (entry 11), which is much smaller than the others. We previously reported that smaller *K*_m_/*K*_i_ values are good parameters for assessing the catalytic activity of the MNP catalysts [54]. For example, the *K*_m_/*K*_i_ values for **10a** and **16f**, both of which function as catalysts are ca. 4.8 and 5.4 (entries 4 and 8), respectively, and these values are close to that for AP (*ca.* 2.3) (entry 2), and smaller than the corresponding values for **16d** (*ca.* 12) and **16i** (*ca.* 20) (entries 6 and 11), whose CTN values are 1.2–1.8.

Another important point is that **16d**, **16f**, **16i**, and **17f** are inhibited by HPO_4_^2−^ in a competitive manner (entries 6, 8, 11, and 18), like that of AP. This type of inhibition is different from our previous supramolecular phosphatases such as **8a** (entry 1) that functions in a single-phase aqueous solution and **10a** that functions in a two-phase solvent system. Based on the above findings, we conclude that the quantitative distribution of **16d**, **16f**, **16i**, and **17f** in the organic phase (Table 1) adequately mimics the binding mode of AP with the substrate and inhibitors (MNP and HPO_4_^2−^, respectively, in this case).

**Figure 11:**
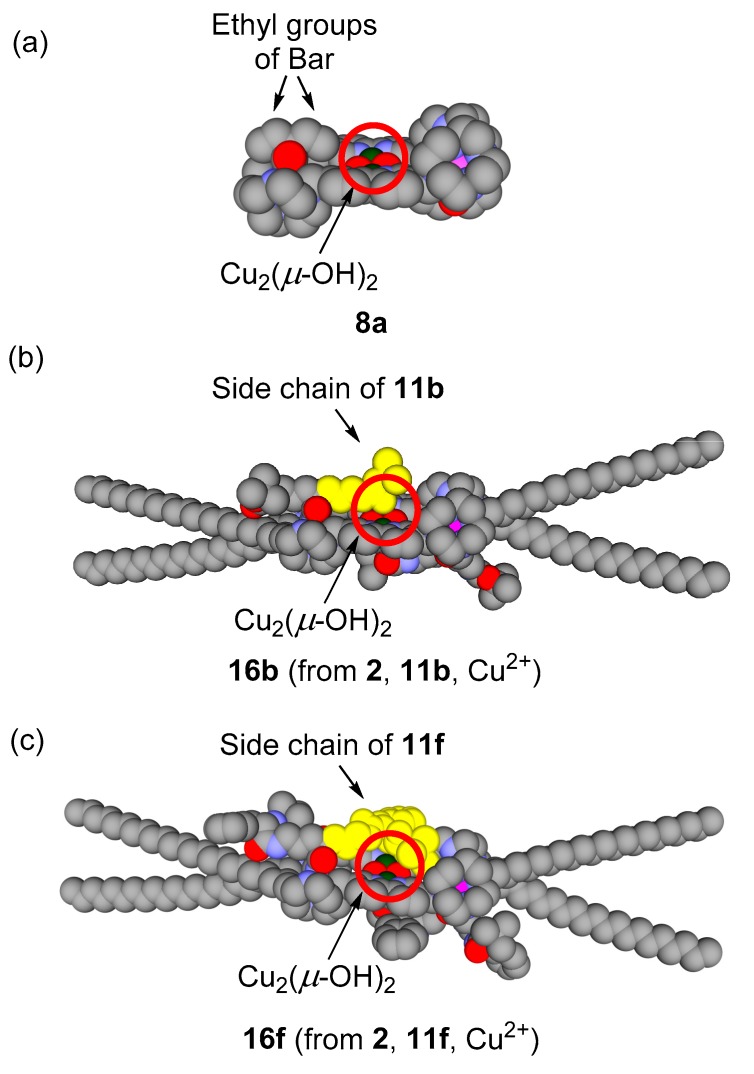
(**a**) X-ray crystal structure of **8a** and (**b** and **c**) proposed structures of **16b** and **16f** (yellow spheres indicate the side chains of the Bar units **11b** and **11f**).

The *K*_m_ values for **16d**–**h** (entries 6–10) (0.3–1.9 × 10^2^ *μ*M) are lower than that for **10a** (entry 4) (3.8 × 10^2^ *μ*M), although the *K*_i_ values for **16d**, **16f**, and **16i** (16, 23, and 0.67 *μ*M, respectively) are also lower than that for **10a** (80 *μ*M). The proposed structure of **16f** generated by BIOVIA Discovery Studio (Ver: 17.2.0) based on the crystal structure of **8a** (Figure 11a, in which Cu^2+^-bound H_2_O molecules are omitted for clarity) [24] suggests that the side chain of the **11f** unit in **16f** (yellow spheres) is located over the Cu_2_(μ-OH)_2_ core, which results in the formation of a hydrophobic pocket similar to the active site of AP, as shown in Figure 11b,c. Therefore, we conclude that the hydrophobicity of supramolecular complexes is important in terms of improving the stabilization of the supermolecule–MNP complexes (i.e., the ES complexes) that have smaller *K*_m_ values and the effective extraction of MNP from the aqueous layer. Namely, the hydrophobic active site of the artificial supramolecular complexes is important for mimicking the hydrophobic active site of natural AP and hydrophobicity/hydrophilicity balance is important for catalytic activity.

## 4. Conclusions

In conclusion, we report on the design, and synthesis of Bar building units that are functionalized with amino acids and related units, with which many supramolecular complexes are formed by the 2:2:2 self-assembly of **2** (Zn_2_L^2^) and Cu^2+^ in a two-phase solvent system based on the structure of the active site of AP. The ease of functionalizing the barbital units allowed us to construct a series of various supramolecular complexes which were then used to assess their catalytic activity for the hydrolysis of MNP and related studies of the reaction mechanism. A comparison of the hydrolysis of MNP in a single-phase system and that in the two-phase system in the presence of **8**, **9**, **15**, or **16** strongly suggest that two-phase solvent system contributes to the improvement in the activity of MNP hydrolysis by reducing the product inhibition by HPO_4_^2−^. In addition, the hydrolysis of MNP by the hydrophobic supramolecular complexes follows Michaelis–Menten kinetics, and functionalization with Boc-protected phenylalanine (**16f**) results in a higher *V*_max_ and a lower *K*_m_, and a greater *k* value than the corresponding values for **9a**. The *K*_m_/*K*_i_ value for **16f** (*ca.* 5.4) is close to that for AP (*ca.* 2.3) and competitive inhibition by inorganic phosphate was observed, unlike for our previously reported complexes such as **10a**–**c**. We therefore conclude that the distribution of the Cu_2_(*μ*-OH)_2_ active site of the supermolecules in both layers in the two-phase solvent system contributes to the improvement of catalytic turnover, and the formation of hydrophobic Cu_2_(*μ*-OH)_2_ site mainly in organic layer appropriately mimics the binding mode of AP with the substrates (for the E–S complexation) and the inhibitors (product inhibition). These findings should be highly useful for the design of more efficient catalysts in reference to biochemistry including enzymatic reactions.

## Figures and Tables

**Table 1:** Distribution of each complex ([**6a**, **13**] = 20 *μ*M in the total solvent including both the aqueous and the CHCl_3_ phase) in the aqueous layer and the organic layer, as estimated by UV/Vis absorption spectra of these complexes in each layer.

-	In H_2_O Layer (%)	In CHCl_3_ Layer (%)
**5a** *^a^*	>98	<2
**6a**	1	99
**7a** *^a^*	35	65
**13d**	2	98
**13f**	4	96
**13i**	<1	>99
**13k**	<1	>99

*^a^* From ref. 54 ([**5a** and **7a**] = 50 *μ*M in total solvent including aqueous and CHCl_3_ phase).

**Table 2:** Kinetics parameters for the hydrolysis of MNP by **8a** and AP in single-phase solvent system (10 mM HEPES buffer (pH 7.4) with *I* = 0.1 (NaNO_3_)) at 37 °C, and **9a**, **10a**, **16b**, **16d**–**n**, **17d**, and **17f** in two-phase solvent system (CHCl_3_/50 mM HEPES buffer (pH 7.4) with *I* = 0.1 (NaNO_3_) (2/8)) at 37 °C.

Entry	Cat. *^a^*	*V*_max_ (*μ*M min^−1^)	*K*_m_ (*μ*M)	*k* (min^−1^) *^b^*	*K*_i_ (*μ*M)	*K*_m_/*K*_i_	CTN *^c^*
1	**8a** *^d^*	(8.9 ± 0.2) × 10^−2 *d*^	(4.1 ± 0.3) × 10^2 *d*^	(2.2 ± 0.2) × 10^−4 *d*^	*ca.* 15 (mixed-type) *^d^*	*ca.* 27	0.4
2	**AP** *^e^*	1.3 ± 0.1 *^e^*	7 ± 4 *^e^*	(2.9 ± 1.8) × 10^−1 *e*^	3 ± 1 (competitive) *^e^*	*ca.* 2.3	>10^3^ *^f^*
3	**9a** *^g^*	(1.4 ± 0.4) × 10^−2 *g*^	(5.4 ± 0.5) × 10^2 *g*^	(2.7 ± 1.0) × 10^−5 *g*^	*ca.* 15 (competitive) *^g,h^*	*ca.* 36	1.0
4	**10a** *^h^*	(6.8 ± 0.3) × 10^−2 *h*^	(3.8 ± 0.2) × 10^2 *h*^	(1.8 ± 0.2) × 10^−4 *h*^	*ca.* 80 (mixed-type) *^h^*	*ca.* 4.8 *^h^*	~4
5	**16b** *^i^*	(3.6 ± 0.2) × 10^−2^	(2.5 ± 0.3) × 10^2^	(1.4 ± 0.3) × 10^−4^	n.d. *^j^*	n.d. *^j^*	2.1
6	**16d** *^i^*	(3.9 ± 0.2) × 10^−2^	(1.9 ± 0.1) × 10^2^	(2.1 ± 0.2) × 10^−4^	16 (competitive)	*ca.* 12	1.8
7	**16e** *^i^*	(2.4 ± 0.2) × 10^−2^	(1.1 ± 0.1) × 10^2^	(2.2 ± 0.4) × 10^−4^	n.d. *^j^*	n.d. *^j^*	2.0
8	**16f** *^i^*	(2.9 ± 0.1) × 10^−2^	(1.2 ± 0.1) × 10^2^	(2.4 ± 0.3) × 10^−4^	23 (competitive)	*ca.* 5.4	2.7
9	**16g** *^i^*	(2.3 ± 0.2) × 10^−2^	(1.0 ± 0.1) × 10^2^	(2.4 ± 0.4) × 10^−4^	n.d. *^j^*	n.d. *^j^*	2.1
10	**16h** *^i^*	(1.2 ± 0.1) × 10^−2^	33 ± 1	(3.6 ± 0.5) × 10^−4^	n.d. *^j^*	n.d. *^j^*	1.1
11	**16i** *^i^*	(8.1 ± 0.2) × 10^−3^	13 ± 1	(6.0 ± 0.4) × 10^−4^	0.67 (competitive)	*ca.* 20	1.3
12	**16j** *^i^*	(1.0 ± 0.1) × 10^−2^	(4.7 ± 0.2) × 10^2^	(2.2 ± 0.2) × 10^−5^	n.d. *^j^*	n.d. *^j^*	1.2
13	**16k** *^i^*	(1.4 ± 0.1) × 10^−2^	(7.6 ± 0.2) × 10^2^	(1.9 ± 0.2) × 10^−5^	n.d. *^j^*	n.d. *^j^*	1.0
14	**16l** *^i^*	(4.5 ± 0.2) × 10^−3^	(3.5 ± 0.1) × 10^2^	(1.3 ± 0.1) × 10^−5^	n.d. *^j^*	n.d. *^j^*	0.6
15	**16m** *^i^*	(7.8 ± 0.3) × 10^−3^	40 ± 1	(2.0 ± 0.1) × 10^−4^	n.d. *^j^*	n.d. *^j^*	1.2
16	**16n** *^i^*	(2.2 ± 0.2) × 10^−2^	72 ± 2	(3.0 ± 0.3) × 10^−4^	n.d. *^j^*	n.d. *^j^*	1.7
17	**17d** *^i^*	(2.5 ± 0.2) × 10^−2^	(1.2 ± 0.1) × 10^2^	(2.2 ± 0.4) × 10^−4^	17 (mixed-type)	*ca.* 7.4	2.0
18	**17f** *^i^*	(2.3 ± 0.2) × 10^−2^	47 ± 2	(5.0 ± 0.6) × 10^−4^	5.7 (competitive)	*ca.* 8.3	2.3

*^a^* Cat = catalyst. *^b^* Calculated by eq. (1). *^c^* Catalytic turnover numbers determined at [MNP] = 1000 *μ*M (see Figure 9). *^d^* From ref. 24 (in a single aqueous solution (10 mM HEPES buffer, pH 7.4 with *I* = 0.1 (NaNO_3_)). *^e^* From ref. 19 (in a single aqueous solution (10 mM HEPES buffer, pH 7.4 with *I* = 0.1 (NaNO_3_)). *^f^* Calculated from data in ref. 19. *^g^* From ref. 53. *^h^* From ref. 54. *^i^* Determined in CHCl_3_/50 mM HEPES buffer (pH 7.4) with *I* = 0.1 (NaNO_3_) (2/8). *^j^* Not determined.
